# Supplementary material for: Fucosylated Chondroitin Sulfates with Rare Disaccharide Branches from the Sea Cucumbers Psolus peronii and Holothuria nobilis: Structures and Influence on Hematopoiesis
Source: Pharmaceuticals (Basel). 2023 Nov 30;16(12):1673. doi: 10.3390/ph16121673 (PMC10748315; doi:10.3390/ph16121673)
Supplement: Supplementary file 1 [file pharmaceuticals-16-01673-s001.zip › pharmaceuticals-2690387-supplementary.pdf]

Supplementary Materials

Fucosylated Chondroitin Sulfates with Rare Disaccharide Branches from the Sea Cucumbers *Psolus peronii* and *Holothuria nobilis*: Structures and Influence on Hematopoiesis

Nadezhda E. Ustyuzhanina, Maria I. Bilan, Natalia Yu. Anisimova, Sofya P. Nikogosova, Andrey S. Dmitrenok, Evgenia A. Tsvetkova, Elena G. Panina, Nadezhda P. Sanamyan, Sergey A. Avilov, Valentin A. Stonik, Mikhail V. Kiselevskiy, Anatolii I. Usov and Nikolay E. Nifantiev

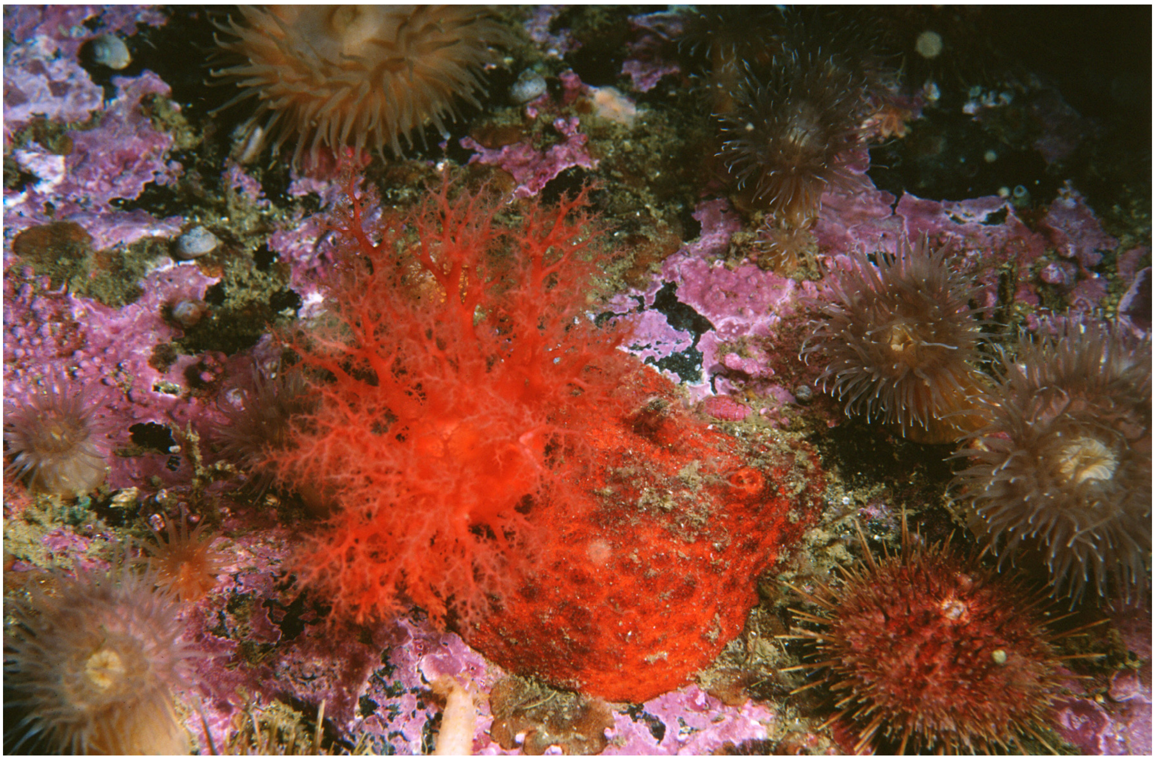

Figure S1. Sea cucumber *Psolus peronii*.

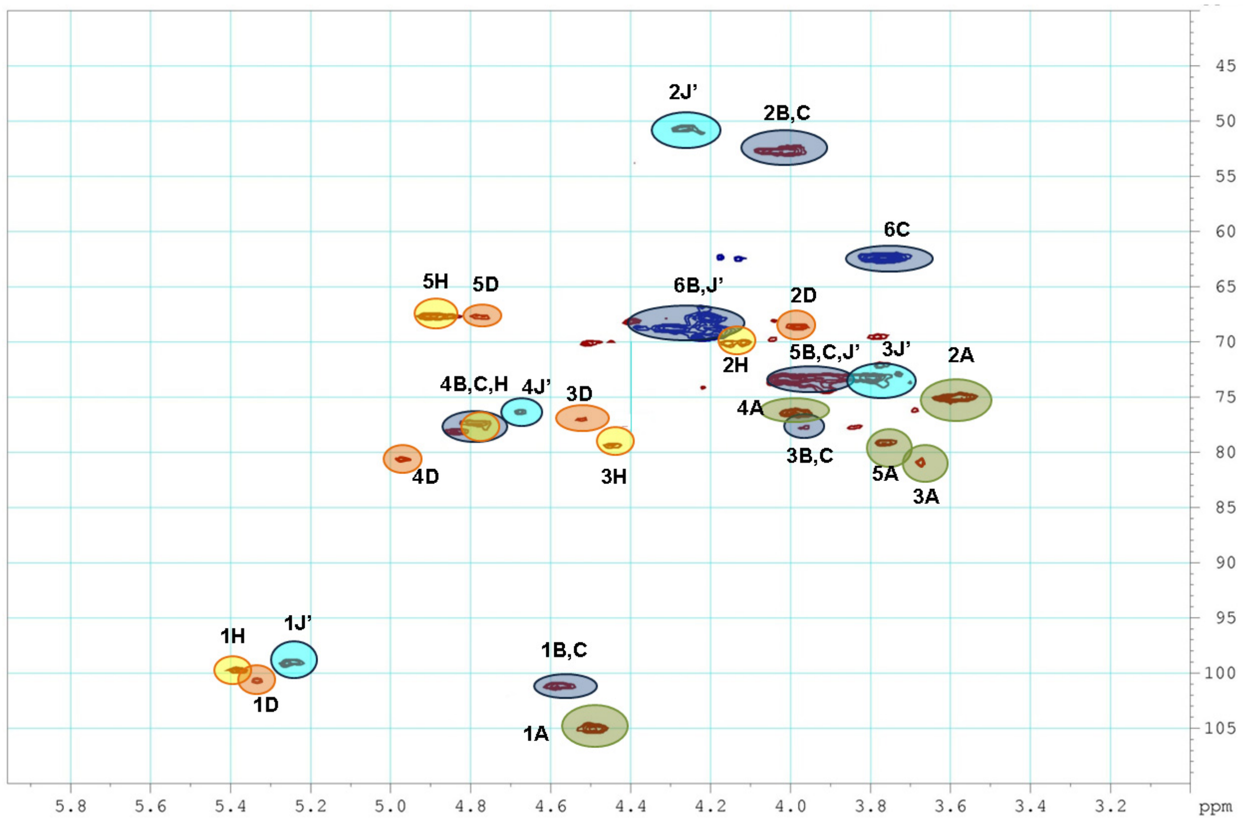

Figure S2. The ^{1}H-^{13}C HSQC NMR spectrum of polysaccharide **PP**.

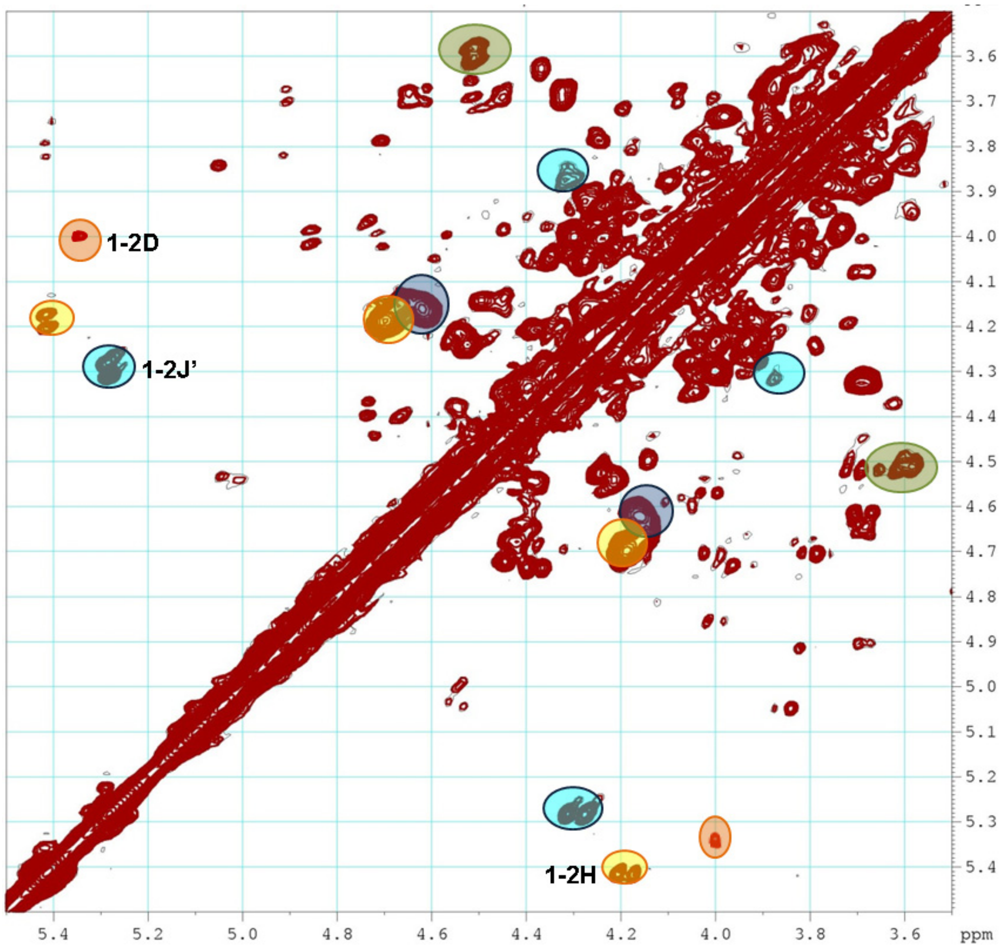

Figure S3. The ^1H-^1H COSY NMR spectrum of polysaccharide **PP**.

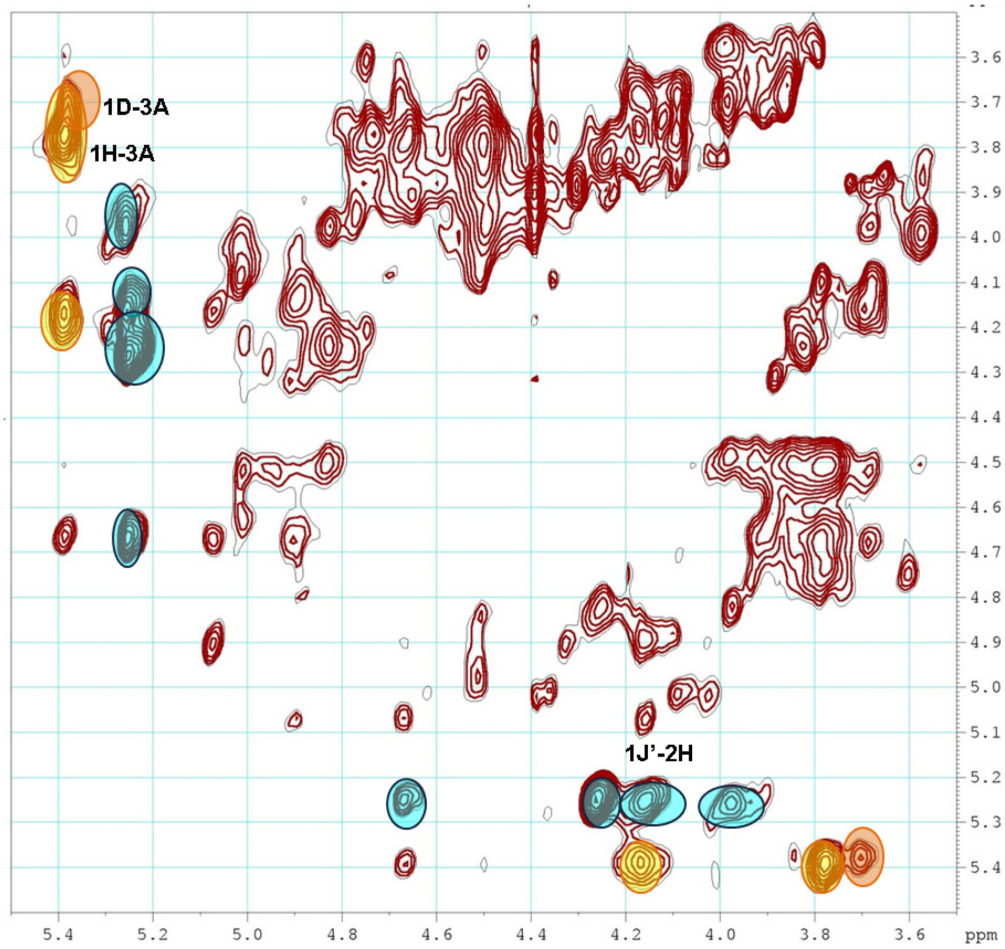

Figure S4. The ^1H-^1H ROESY NMR spectrum of polysaccharide **PP**.

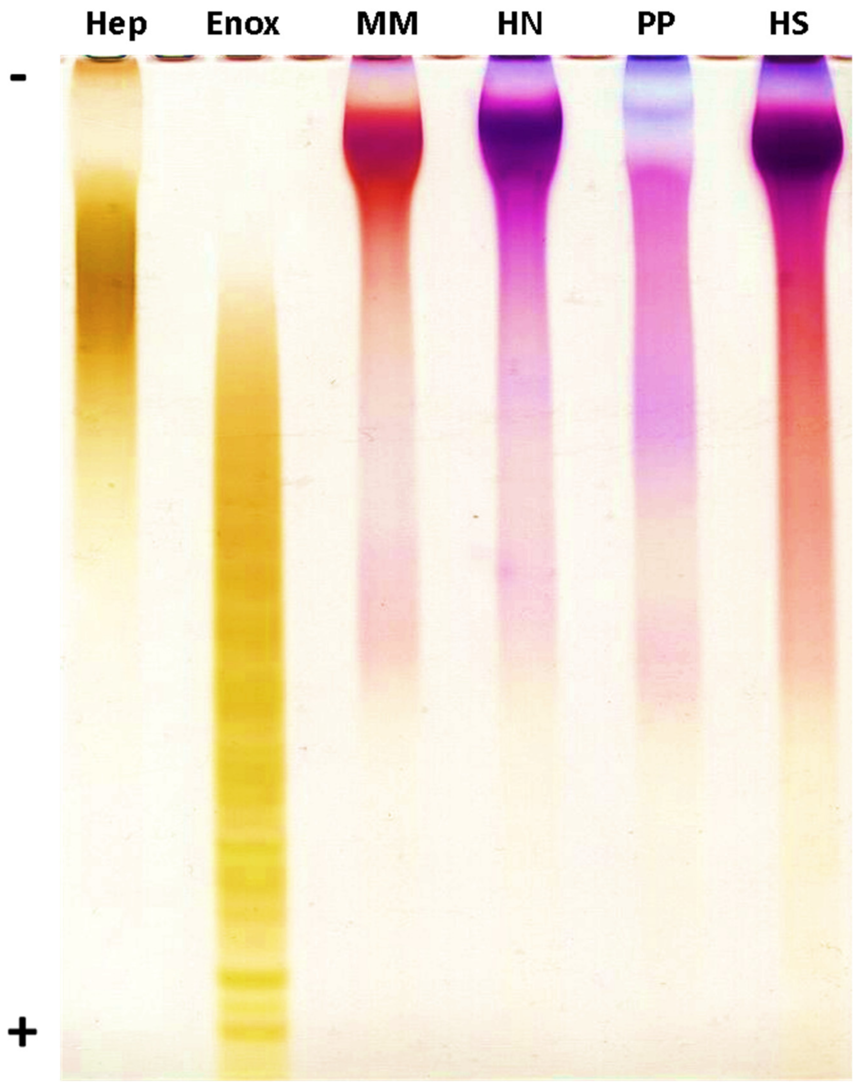

Figure S5. Electrophoresis in polyacrylamide gel. Hep – heparin (Sigma), Enox – enoxaparin (Clexane®, Sanofi), **MM** – FCS from *Massinium magnum* [29], **HN** – FCS from *Holothuria nobilis*, **PP** – FCS from *Psolus peronii*, **HS** – FCS from *Holothuria spinifera* [30].

Table S1. The data of the ^{1}H and ^{13}C NMR spectra (chemical shifts, ppm) of fucosylated chondroitin sulfates **HN** and **PP**.

Residue	H-1<br>(C-1)	H-2<br>(C-2)	H-3<br>(C-3)	H-4<br>(C-4)	H-5<br>(C-5)	H-6<br>(C-6)
**A**→4)-β-D-Glc*p*A-(1→	4.48	3.58	3.68	4.00	3.71	-
	(105.0)	(75.0)	(80.7)	(76.6)	(78.2)	(176.0)
**B**→3)-β-D-Gal*p*NAc4*S*6*S*-(1→	4.58	4.07	3.95	**4.81**	4.00	**4.33, 4.20**
	(100.9)	(52.7)	(77.9)	**(77.2)**	(73.2)	**(68.5)**
**C**→3)-β-D-Gal*p*NAc4*S*-(1→	4.58	4.07	3.95	**4.81**	4.02	3.81
	(100.9)	(52.7)	(77.9)	**(77.2)**	(76.2)	(62.3)
**D** α-L-Fuc*p*3*S*4*S*-(1→	5.34	3.95	**4.53**	**5.01**	4.85	1.37
	(100.5)	(67.6)	**(76.6)**	**(80.6)**	(67.6)	(17.2)
**G** →2)-α-L-Fuc*p*3*S*-(1→	5.37	4.18	**4.53**	3.91	4.97	1.32
	(98.4)	(70.0)	**(78.7)**	(69.9)	(67.7)	(16.8)
**H** →2)-α-L-Fuc*p3S*4*S*-(1→	5.39	4.18	**4.53**	**4.78**	4.97	1.31
	(99.5)	(70.0)	**(78.7)**	**(77.9)**	(67.7)	(16.8)
**J** α-D-Gal*p*NAc4*S*6*S*-(1→	5.27	4.28	3.79	**4.68**	4.00	**4.33, 4.20**
	(99.0)	(50.2)	(75.8)	**(79.2)**	(73.4)	**(68.5)**
**J'** α-D-Gal*p*NAc4*S*6*S*-(1→	5.25	4.25	3.78	**4.67**	4.00	**4.33, 4.20**
	(98.9)	(50.4)	(73.2)	**(76.2)**	(73.3)	**(68.5)**

Table S2. Statistical significance of the difference of soluble cytokine level in a growth medium after incubation of mouse bone marrow cells with studied compounds: **HN, MM, HS, PP**, r G-CSF and LPS comparing to a Control or r G-CSF effect.

**Group**	**IL-6**		**IL-2**		**TNF**		**GM-CSF**	
	vs Control	vs G-CSF	vs Control	vs G-CSF	vs Control	vs G-CSF	vs Control	vs G-CSF
**Control**	-	0.000	-	0.180	-	0.000	-	0.000
**r G-CSF**	0.000	-	0.180	-	0.000	-	0.000	-
**LPS**	0.000	0.000	0.006	0.195	0.000	0.000	0.000	0.032
**HN**	0.000	0.014	0.192	0.178	0.000	0.104	0.000	0.007
**MM**	0.000	0.125	0.115	0.439	0.002	0.012	0.000	0.000
**HS**	0.000	0.012	0.144	0.236	0.000	0.000	0.000	0.453
**PP**	0.000	0.006	0.248	0.221	0.000	0.076	0.000	0.001
